# The complete chloroplast genome of *Piptanthus nepalensis*, a medicinal plant

**DOI:** 10.1080/23802359.2021.1994897

**Published:** 2022-05-09

**Authors:** Huigai Sun, Kaiyan Zheng, Xiaowei Han, Junna Song, Yang Li, Lei Du, Yuguang Zheng, Donglai Ma

**Affiliations:** a Traditional Chinese Medicine Processing Technology Innovation Center of Hebei Province, Hebei University of Chinese Medicine, Shijiazhuang, China; b International Joint Research Center on Resource Utilization and Quality Evaluation of Traditional Chinese Medicine of Hebei Province, Shijiazhuang, Hebei, China

**Keywords:** Phylogeny, chloroplast genome, *Piptanthus nepalensis*, Leguminosae

## Abstract

*Piptanthus nepalensis* (Hooker) Sweet is a semi deciduous or deciduous shrub belonging to the genus *Piptanthus,* Leguminosae. *P. nepalensis* has been used as a folk medicinal herb in Nepal and was cultivated all over the world as an ornamental plant. In the present study, we sequenced the entire genome of the chloroplast of *P. nepalensis*. The total length of the chloroplast genome in *P. nepalensis* is 152,195 bp, including a large single-copy region of 82,048 bp, a small single-copy region of 17,675 bp, and a pair of inverted repeats regions of 26,236 bp. The overall guanine-cytosine (GC) content of the genome was 36.7%. There are 131 genes in the chloroplast genome of *P. nepalensis*, including 85 protein-coding genes, 8 rRNA genes and 38 tRNA genes. Phylogenetic analysis showed that *P. nepalensis* is closely related to *Maackia floribunda*.

*Piptanthus nepalensis* (Hooker) Sweet, commonly known as ‘Evergreen Laburnum,’ is a semi deciduous or deciduous shrub belonging to the genus *Piptanthus,* Leguminosae. This shrub is mainly distributed in Nepal, India, Myanmar, China Tibet, East Himalaya and West Himalaya. *Piptanthus nepalensis* has been used as an ethnomedicinal plant in Nepal and its crude extract showed antibacterial property against some gram-positive test bacteria and gram-negative bacteria (Martini et al. 1990; Bhattarai and Basukala 2016). *Piptanthus nepalensis* is s bushy shrub with leaves composed of 3 dark green leaflets, and short racemes of yellow flowers in late spring and early summer, followed by flat green pods, and it has been cultivated all over the world as an ornamental plant.

Chloroplast genome is very conservative in the organizational structure and gene content, and this feature makes the chloroplast genome an ideal tool for studying plant evolution and phylogenetics (Jansen et al. 2007). In the present study, we sequenced and assembled the chloroplast genome of *P. nepalensis* and analyzed its phylogenetic position. The leaves of *P. nepalensis* were collected from Gongbujiangda County, Tibet Autonomous Region, China (29°53′17.12″N, 93°14′41.34″E) and deposited in the Herbarium of School of pharmacy, Hebei University of Chinese Medicine, under the voucher number 20181022-03. Genomic DNA was extracted from the leaves of *P. nepalensis* using Plant Genomic DNA Kits (Tiangen Biotech Co., Beijing, China). Illumina HiseqX TEN platform was used for DNA sequencing. Approximately 10 GB clean reads (paired-end 150 bp) were generated and used for chloroplast genome assembly using NOVOPlasty v 3.7.2. (Dierckxsens et al. 2017). The assembled chloroplast genome was annotated with PGA (Qu et al. 2019). The *P. nepalensis* chloroplast genome sequence was deposited in GenBank (Accession number: MN841455).

The complete length of the chloroplast genome of *P. nepalensis* is 152,195 bp, with a canonical quadripartite structure, consisting of a large single-copy region of 82,048 bp, a small single-copy region of 17,675 bp, and a pair of inverted repeats regions of 26,236 bp. The overall guanine-cytosine (GC) content of the genome was 36.7%. A total of 131 genes were annotated from the chloroplast genome of *P. nepalensis*, including 85 protein-coding genes, 8 rRNA genes, and 38 tRNA genes.

To understand the evolutionary status of *P. nepalensis*, 11 chloroplast genomes of related plant species were downloaded from NCBI and phylogenetic analysis were conducted based on the full sequences of the 12 chloroplast genomes using maximum likelihood method. The chloroplast genome of *Robinia pseudoacacia* was used as the outgroup and the phylogenetic tree was reconstructed using MAGE-X (Kumar et al. 2018). Phylogenetic analysis showed that the *P. nepalensis* and *Maackia floribunda* were closely clustered into a clade (Figure 1). The chloroplast genome of *M. amurensis* provides useful information for the further study of the genetic diversity and ecological protection of Species of Sophoreae, Leguminosae.

## Data Availability

The genome sequence data that support the findings of this study are openly available in GenBank of NCBI at (https://www.ncbi.nlm.nih.gov/) under the accession no. MN MN841455. The associated BioProject, SRA, and Bio-Sample numbers are PRJNA685302, SAMN17082319, and SRR13263232, respectively. The phylogenetic tree of *P. nepalensis* and the other 11 plant species. The external group was *Robinia pseudoacacia*. The numbers on the branches represent the confidence in the relationship between the two species.
